# Medium Access Control Protocols for Cognitive Radio Ad Hoc Networks: A Survey

**DOI:** 10.3390/s17092136

**Published:** 2017-09-16

**Authors:** Mahdi Zareei, A. K. M. Muzahidul Islam, Sabariah Baharun, Cesar Vargas-Rosales, Leyre Azpilicueta, Nafees Mansoor

**Affiliations:** 1 Tecnologico de Monterrey, Escuela de Ingeniería y Ciencias; Monterrey 64849, Mexico; m.zareei@ieee.org (M.Z.); leyre.azpilicueta@itesm.mx (L.A.); 2 Department of Computer Science and Engineering, University of Liberal Arts Bangladesh (ULAB); Dhaka 1209, Bangladesh; muzahidul.islam@ulab.edu.bd (A.K.M.M.I.); nafees@ieee.org (N.M.); 3 Malaysia-Japan International Institute of Technology (MJIIT), Universiti Teknologi Malaysia (UTM); Kuala Lumpur 54100, Malaysia; sabariahb@utm.my

**Keywords:** cognitive radio ad hoc network, medium access control, cognitive radio, spectrum mobility, spectrum sensing, spectrum sharing

## Abstract

New wireless network paradigms will demand higher spectrum use and availability to cope with emerging data-hungry devices. Traditional static spectrum allocation policies cause spectrum scarcity, and new paradigms such as Cognitive Radio (CR) and new protocols and techniques need to be developed in order to have efficient spectrum usage. Medium Access Control (MAC) protocols are accountable for recognizing free spectrum, scheduling available resources and coordinating the coexistence of heterogeneous systems and users. This paper provides an ample review of the state-of-the-art MAC protocols, which mainly focuses on Cognitive Radio Ad Hoc Networks (CRAHN). First, a description of the cognitive radio fundamental functions is presented. Next, MAC protocols are divided into three groups, which are based on their channel access mechanism, namely time-slotted protocol, random access protocol and hybrid protocol. In each group, a detailed and comprehensive explanation of the latest MAC protocols is presented, as well as the pros and cons of each protocol. A discussion on future challenges for CRAHN MAC protocols is included with a comparison of the protocols from a functional perspective.

## 1. Introduction

The current wireless networking paradigm consists of the connectivity of many heterogeneous devices through links operating under dynamic environments. These devices will demand an increasing usage of communication resources such as spectrum, and it will not be possible to handle the increasing demand unless automatic administration and protocols without human intervention are used. Moreover, with the new trends in wireless devices and services, the spectrum scarcity problem has become one of the main research focuses [1,2,3]. Therefore, today’s wireless communication networks encounter difficulties on the efficient management of this increasing complexity. The answer for an efficient use of scarce communication resources is the use of multiple access protocols that maximize the number of users and throughput. Although protocols at the MAC layer have been studied for many years, their use in new communication paradigms, e.g., Cognitive Radio (CR), requires modifications that need to be identified. This paper provides a survey in detail of the state-of-the-art in MAC protocols especially for CR networks.

In 2000, the Cognitive Radio Network (CRN) paradigm was proposed by Mitola [4], which uses cognitive processes that can observe the current state of the network and then decide, act and plan based on those observations. CR became a favorable concept to overcome inefficiency in spectrum usage, both in unlicensed and licensed bands [5,6,7,8,9]. Meanwhile, static spectrum allocation in wireless services causes temporal and geographical holes in the spectrum in authorized bands [10,11]. To utilize the unoccupied band and to resolve the spectrum scarcity problem, the Federal Communications Commission (FCC) has decided to allow the coexistence of unlicensed users defined as Secondary Users (SUs) and licensed users named Primary Users (PUs), through the opportunistic use of spectrum holes without hindering the performance of the PUs [12,13].

Currently, there are several ongoing CR-related standardization efforts by the IEEE 802 community like the IEEE 802.22 [14], IEEE 802.11af [15] and also IEEE 1900 [16]. In cognitive radio networks, SUs are equipped with one or more Cognitive Radios (CRs) to enable them to sense the presence of PUs and opportunistically tune to the spectrum band, which is not in use by PUs. As soon as a particular PU starts its activity in a certain channel, the SUs have to vacate that channel and move between unused spectra to avoid interference with PUs. CRN can be divided into two categories based on the network architecture, namely the infrastructure-based CR network and Cognitive Radio Ad Hoc Networks (CRAHNs) [17]. The first category uses a central entity, such as a base station (similar to a cellular network) in order to manage and control the network [18,19]. Whereas CRAHNs do not rely on any predefined infrastructure backbone, instead, the communication between CR users takes place in an ad hoc manner [20]. As CRAHN has a lower cost of implementation, lower system complexity and faster align positioning compared to infrastructure-based CRN, it is a more suitable option.

In recent years, several MAC protocols for CR have been proposed. To assess and review the existing work, a few surveys have been published. In [21], MAC protocols are divided based on control information exchange into four groups and later compared with each other using an extended framework proposed in [22]. Yao et al. [23] categorized multi-channel MAC protocols based on the channel negotiation mechanism and discussed their characteristics, as well as highlighted their necessary modifications to operate in an opportunistic manner. Wang et al., in 2008 [24], discussed the differences between MAC protocols for CR and traditional multi-channel protocols. A survey on Opportunistic Spectrum Access (OSA) networks presented in [25] classifies the network into distributed and centralized, and several CR MAC protocols were analyzed and reviewed. In 2009, Claudia et al. presented in [26] a survey on MAC protocols specifically for CRNs, where they consider both centralized and ad hoc networks at the same time. Similarly, a more recent study by Domenico et al. provided in [27] a comprehensive review on MAC protocols for CRN considering both centralized and decentralized networks.

As can be observed from existing surveys, most of the concentration is given to spectrum management functionalities, and less attention is given to the MAC protocols, especially for decentralized networks, concluding that there is no comprehensive survey paper on MAC protocols for CRAHN. Thus, this paper anticipates to provide a comprehensive and intensive study by giving a detailed summary of the state-of-the-art in MAC layer protocols with a concentration on CRAHN. Before surveying MAC protocols, we present a brief description of CR fundamentals and explain each cognitive radio function. Later, the MAC protocols are divided into three groups based on their access scheme where the salient features of MAC protocols are presented, and the pros and cons of each protocol are discussed and highlighted. Based on the analytical study of the existing MAC protocols, we have provided a discussion on the open research topics to be investigated more deeply in the future. Moreover, the comparison between protocols is introduced in a table to give the reader a broader view of all of the protocols. Thus, the contributions are the following:

- It provides a comprehensive survey of the CR spectrum functions of sensing, decision, sharing and mobility together with their connection to MAC protocols.
- It reviews the state-of-the-art MAC protocols in CRAHN. The pros and cons of each protocol are discussed, and the performance issues are highlighted.
- It highlights open issues and challenges that need to receive more attention in the current literature as the future research direction.

The rest of the paper outline is as follows: Section 2 presents the CR fundamental concepts, where the different components of the cognitive cycle are explained further. A detailed review of CRAHN MAC protocols is discussed in Section 3, where the pros and cons of each protocol are highlighted. In Section 4, we introduce a discussion on open challenges that have not been thoroughly explored. Finally, conclusions are given in Section 5, as well as a discussion of open issues and design considerations for future MAC protocols for CRAHN.

## 2. Cognitive Radio Networks

In contrast to classical ad-hoc networks, CRAHNs have some key distinguishing factors. The most important factor is changing the spectrum environment over an ample frequency range, varying over time and space. Classical ad hoc networks use pre-chosen channels that stay unaltered for a duration of time [28]. This also creates a problem for CRAHNs to control the topology of the network. In traditional ad hoc networks, topology control has been done through the use of beacons [29]. Though, in CRAHNs, due to a large range of possible spectrum, this is not practical, because it is more likely that CRAHNs have incomplete topology information. Moreover, being multi-hop and multi-channel make the arrangement of routing in CRAHNs a very challenging task compared to the classical ad hoc networks [30,31,32].

A CR device must be able to collect information on the spectrum usage in its surroundings to know the set of available channels to be used for communications in an opportunistic way. A cognitive user can only access the idle frequency band, and when a signal from a PU is detected, the cognitive user must vacate the allocated spectrum in order to avoid the generation of interference to PUs, this is called spectrum mobility. As the control and management of communication over wireless channels occur at the MAC layer, other relevant tasks for CRs as sensing the channel, spectrum sharing, resource allocation and spectrum handoffs become essential for connectivity.

Since there is no central entity for the operation of ad hoc networks, the previously-mentioned MAC functionalities impose an extra challenge on ad hoc networks. For example, spectrum sensing in CRAHNs is not controlled and synchronized by the focal network element, but by each user, i.e., in a distributed way. Thus, in CRAHNs, the need for cooperation with neighboring nodes for distributed functionality (e.g., spectrum sensing, sharing and access) is increased.

### 2.1. Cognitive Radio Functions

**Figure 1:**
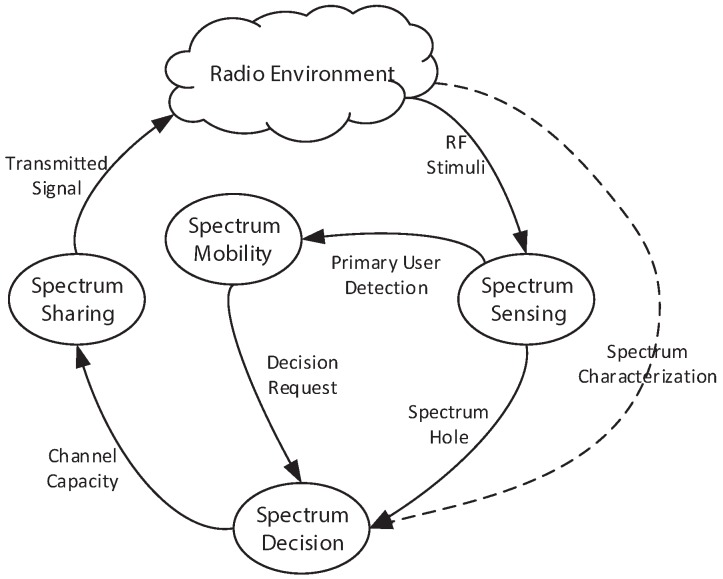
The cognitive cycle.

**Figure 2:**
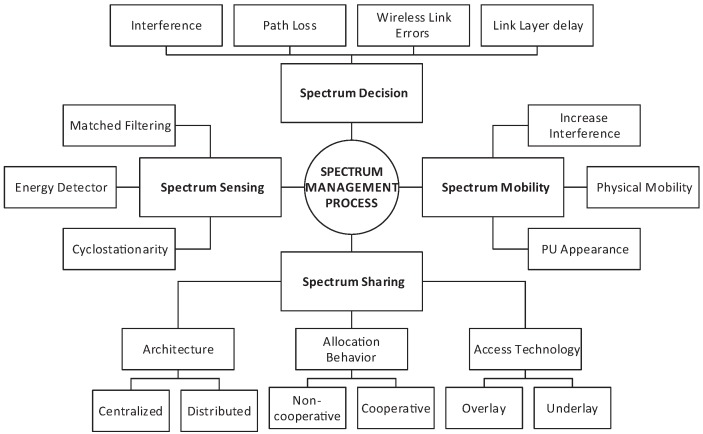
Spectrum management process.

Four of the most important CR functions for spectrum control are: spectrum mobility, spectrum sensing, spectrum sharing and spectrum decision [17]. Figure 1 shows the cognitive cycle, and Figure 2 shows the spectrum management process with the functionalities that are discussed in this paper.

#### 2.1.1. Spectrum Sensing

Identifying available spectrum using spectrum sensing algorithms is a fundamental functionality in CR networks. By providing CR with the spectrum sensing information, CR becomes aware of the adjustments in its encompassing surroundings and can adapt itself to those changes. The research community has proposed different techniques for spectrum sensing to identify and detect a signal transmission. These techniques can be grouped into three categories; namely, matched filter coherent detection [33,34], energy detection [35,36,37] and cyclostationary feature detection [38,39]. Energy detection is the technique most used because it presents low computational and implementation complexities. Assume that in a communications channel, a transmitted signal from a PU, s(n), experiences Additive White Gaussian Noise (AWGN) w(n), where *n* is the sample index, then the received signal by an SU, y(n), has the following form:

(1) y(n)=s(n)+w(n)

Note that when the PU is not transmitting, s(n)=0. For accurate spectrum sensing, CR devices have to measure the power of the PUs signal alone [40]. Therefore, channel estimations must be obtained when other CR devices are not transferring, which requires coordination between CR users. Achieving this coordination is a difficult task due to the lack of synchronization in the network and the relative positions and separation distances among CR devices. In addition to the coordination, the definition of the times for sensing and transmission is an essential trade-off between guaranteeing protections to the PUs and expanding the information throughput at the MAC layer. Spectrum sensing is firmly identified with other range administration capacities and additional layering protocols (PHY and MAC) to give data on spectrum accessibility [41].

Each individual node experiences many factors affecting its performance for the spectrum sensing functions, such as shadowing, fadings due to multipath effects and the receiver uncertainty problem [42,43]. However, to overcome unreliable observations from individual nodes, cooperative decision making with observations collected distributively in space of each individual node can be pursued [44,45]. This is usually referred to as the cooperative spectrum sensing method, which combines the spectrum information from local sensing of multiple CRs in order to minimize the sensing error. There are several works in the literature that show how cooperation can improve the performance of the overall spectrum sensing and maximize the probability of detecting the PU [46,47]. Cooperative sensing has also been proven to overcome and mitigate the problem of shadowing, fading and the receiver uncertainty problem.

In cooperative spectrum sensing, each node needs to sense the channel and later report the information to a central entity or share it with its neighbors. Considering the multipath propagation environment between the PU and CRs, fading in sensing channels is usually Rayleigh distributed with AWGN. Usually, the channel used by the nodes to report the sensing information is assumed to be error-free, [48,49,50]. However, in practice, as the reporting channel also has noise and interference, the common receiver receives the cooperative decisions with error.

#### 2.1.2. Spectrum Decision

Once the accessible spectrum bands and the associated potential estimated channel capacity are identified, according to their Quality of Service (QoS) requirements, appropriate operating spectrum bands can be selected [51,52]. Before selecting the appropriate spectrum band, the CR nodes need to know issues such as interference, the characteristics of path loss, the amount of wireless link errors and delay at the link layer based on the local observations and the primary network information. In CRAHN, the spectrum result includes mutual responsibility of spectrum selection and the route establishment.

#### 2.1.3. Spectrum Sharing

Since there might be different CR nodes attempting to get to the spectrum, the spectrum sharing functionality coordinates CR users’ transmissions to keep different users from crashing in covering parts of the spectrum [53]. Channel allocation does not only depend on spectrum availability, but in order to reduce interference with PU users, it is also determined based on existing spectrum access policies. It also requires at the link layer spectrum awareness together with sensing to achieve an intelligent packet scheduling scheme [54]. As is shown in Figure 2, spectrum sharing can be classified based on different parameters. The spectrum sharing architecture can be categorized into centralized and distributed, where in centralized, the spectrum allocations are controlled by one entity, whereas in the distributed architecture (applied in CRAHN), each individual node can access a spectrum based on a local policy. Spectrum can be allocated cooperatively or non-cooperatively. In cooperative allocation, interference measurement of each node is considered, whereas in the non-cooperative method, only a single node measurement is considered. Based on the access technology, spectrum sharing can be categorized into overlay and underlay spectrum sharing. In the overlay scenario, the unused part of the spectrum is considered for SUs’ transmission, whereas in underlay, SUs transmission is considered as noise for the PUs’ signal.

#### 2.1.4. Spectrum Mobility

The event of spectrum handoff or mobility is when the conditions of the channel in use by the CR node degrade due to interference or outage in the channel or node movement or PU appearance, and the CR node must change its frequency of operation so that the communication continues in a different free part of the spectrum [55]. The different layer protocols of the network stack, such as protocols at the transport layer closely coupled with the lower level spectrum detecting, neighbor disclosure in a link layer and routing protocol, need to adapt to the channel transmission parameters to execute spectrum handoffs efficiently [42,55].

### 2.2. Mobility Perturbs in CRAHN

In CRAHN, node mobility can change the network topology, disrupt the steady state of the network and become a major issue that needs to be addressed. Moreover, as the network topology changes due to node mobility, it affects the link density and stability of the communication between a node and its neighbors. There are several parameters that can be considered for measuring the link stability such as link transmission quality or lifetime of the link [56]. Since the links with the neighbors are the basic building block of a CRAHN, the lack of link stability can hinder transactions at higher layers.

Mobility in CRAHN can occur in two ways, namely spectrum mobility and user mobility, where both can affect the link stability [42,57,58]. In spectrum mobility, CR users have to vacate the frequency in use to avoid causing interference with PUs. User mobility refers to changes in the physical locations of nodes, CR user or PUs [59], which result in a changing network topology [60]. Since a network consists of mobile nodes, as well as static nodes, maintaining a connection between nodes would be a challenging task. Moreover, since nodes change physical locations, spectrum handoffs may also follow. Thus, for designing a mobility management scheme for CRAHN, both types of mobilities must be carefully considered. A self-organizing and self-correcting CRAHN needs to mitigate the effects caused by mobility.

## 3. Survey of State-Of-The-Art MAC Protocols

Traditionally, the MAC sub-layer is at the link layer, where the link layer is in charge of the communication between adjacent nodes. Therefore, the purposes of the CRAHN MAC protocol not only include the enhancement of channel use and throughput, but also include the mechanism of spectrum controlling modules; for example, spectrum sharing and spectrum access tasks to find the technique for data transmissions [61,62]. Thus, an efficient MAC protocol must address the sensing, access and mobility of the spectrum, which are the main functions of the cognitive cycle.

**Figure 3:**
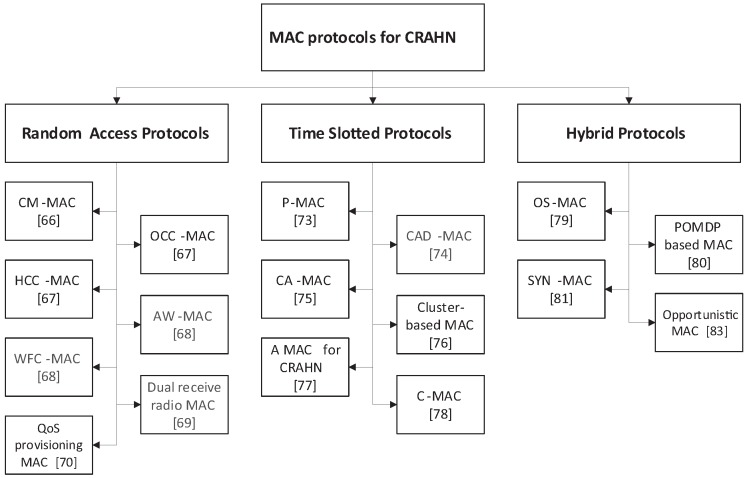
Classification of the MAC protocols studied.

In the following subsections, the existing MAC protocols are categorized based on their medium access scheme; these schemes are random access, time-slotted and hybrid (combination of random access and time-slotted schemes). In this section, the popular approaches in each scheme are introduced, and the advantages and disadvantages of each protocol are discussed. Figure 3 shows the MAC protocols that are considered in this paper.

### 3.1. Random Access Protocols

Random access protocols arise from protocols such as Carrier Sense Multiple Access (CSMA) where the use of the communication channel is permitted to a random number of nodes without central coordination. This group of protocols is also known as Carrier Sense Multiple Access/Collision Avoidance (CSMA/CA). The advantage of CSMA-based protocols is that resources are allocated on demand, making it easily adaptable to traffic fluctuations and protocol changes.

There are several random access MAC protocols proposed for CRAHN where each uses different approaches to improve network performance. For example, the hardware-constrained MAC protocol [63] tries to simplify the hardware requirement by considering the hardware constraint. The Dynamic Open Spectrum Sharing (DOSS) protocol [64] tries to overcome the exposed node and hidden node problems. Dynamic channelization proposed in the Single Radio Adaptive Channel (SRAC) [65] combines frequencies based on the requirements of the CR user. Distributed Channel Assignment (DCA) uses spectrum pooling to increase spectral efficiency as an extension of IEEE 802.11 CSMA/CA. In the remainder of this section, we take a closer look at some of the proposed random access-based MAC protocols for CRAHN.

#### 3.1.1. MAC Protocol with Mobility Support

The protocol in [66] is a variation of CSMA/CA that helps CR nodes adapt to the conditions of their surrounding vicinity to a Primary Exclusive Region (PER) by using the Radio Signal Strength Indicator (RSSI). The protocol also supports mobility. It utilizes a Common Control Channel (CCC) as a part of the request to trade the control frame, for example Ready-to-Send (RTS), Clear-to-Send (CTS) and Acknowledgment (ACK) frames. CM-MAC uses Transmitter Spectrum Sensing (TSS) and Receiver Spectrum Sensing (RSS). TSS is performed by the CR transmitter, while RSS by the receiver side. After performing the sensing, the spectrum sensing data are included in the CTS/RTS frames, where each CR node can obtain the spectrum accessibility information of one hop from broadcast RTS/CTS frames. The sensing information of TSS/RSS that is integrated into RTS/CTS/ACTS can be received by all of the neighboring CRs. Thus, this process is sufficient to ensure nodes have the necessary information for transmitting packets successfully to the next hop. After selecting the accessible channel set, to improve the throughput, CM-MAC transfers the split data payload simultaneously on multiple channels.

#### 3.1.2. Cross-Layer MAC for Congestion-Contention and Power Control

The optimal Cross-Layer Cognitive MAC Protocol (OCC-MAC) [67] proposed for multi-hop CRAHNs is a slotted random access MAC protocol that employs the OSA concept. It also uses slotted *p*-persistent CSMA to control replaced messages through the CCC. In OCC-MAC, persistence probability and the transmission power are balanced on the premise of the entrance rate controlled by sources to expand the aggregate net income of the auxiliary framework while the impact probability to the PU is kept lower than the allowable threshold. OCC-MAC divides time into fixed length intervals. CR nodes synchronize and they use a predefined slot at the beginning of each time.

In Heuristic Cross-Layer Cognitive MAC (HCC-MAC) [67], SUs do not interchange control data through any of the cognitive radios; therefore, one of their cognitive radios is used for eavesdropping on current communications (idle, success or collision) inside each band. To utilize the spectrum opportunity, both HCC-MAC and OCC-MAC try to balance the contention and interference level among the cognitive links. However, due to overhead blockage in the network and the long queue of unsuccessful control messages in CCC, the OCC-MAC is not a suitable option to implement in practice.

#### 3.1.3. Adaptive Power-Controlled MAC Protocols

In [68], the authors present an optimal centralized algorithm based on the bipartite matching technique for ideal channel assignment and control provision problems. The key objective is to maximize capacity as seen by simultaneous CR transmissions and to maximize throughput. Access Windows MAC (AW-MAC) is a centralized polynomial-time algorithm, which is a CSMA-based MAC protocol proposed for single-hop CRN.

**Figure 4:**
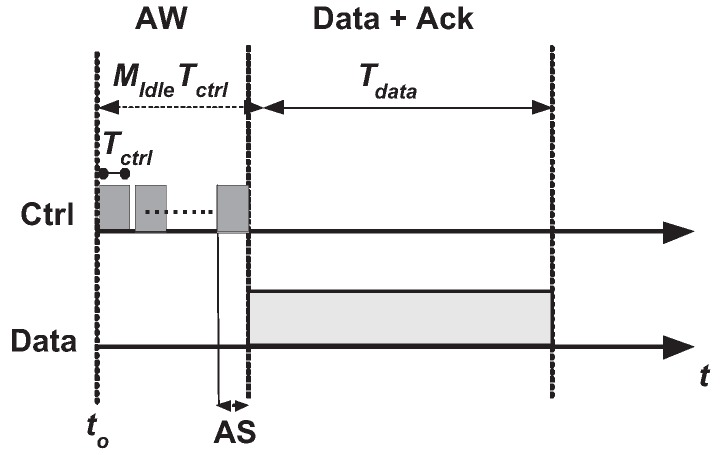
AW-MAC basic operation.

An efficient circulated channel task for a multi-hop CRN has been proposed by the authors that depends just on data given by the two imparting CR users. Utilizing an agreeable task among neighboring CR users, the proposed distributed scheme improves the CRN throughput. Figure 4 shows AW-MAC basic operation framework. The Worst Feasible Channel MAC (WFC-MAC) is a protocol based on CSMA for multi-hop CRNs, which is based on the distributed scheme. Every node has a solitary half-duplex radio that has a capacity of “dual-receive single-transmit”; therefore, each radio transmits over one channel at a time, but can get two autonomous bits over any two of the accessible channels, simultaneously.

#### 3.1.4. Single-Transmit, Dual-Receive Radios

Similarly to the WFC-MAC protocol, the authors proposed a distributed contention-based MAC protocol for multi-hop ad hoc networks, which uses the “dual-receive” capability of radios [69]. The main design goal of this protocol is to maximize network throughput by allowing several simultaneous transmissions to perform at the highest possible rate. A common channel has been assigned for control purposes. Nodes not transmitting always have one of their two receivers listening to the CCC, while receiving data over the other chain can alleviate the hidden terminal problem in the multi-channel scenario.

To decrease the possibility of collision, every CR node *i* keeps up a list of channels that are available (${\mathrm{AC}}_{i}$) and a list of nodes that are busy (${\mathrm{BN}}_{i}$). ${\mathrm{AC}}_{i}$ comprises channels that are not occupied and are free to use. ${\mathrm{BN}}_{i}$ comprises the identification of nodes currently transmitting or getting information bundles in the area of node *i*. Imagine a scenario where CR *i* has an information bundle to transmit to CR *j*. First, it checks ${\mathrm{AC}}_{i}$ and ${\mathrm{BN}}_{i}$; if *j*∈ ${\mathrm{BN}}_{i}$ and ${\mathrm{AC}}_{i}$ ≠∅, node *i* fights over the CCC using a CSMA/CA-type protocol. After getting an RTS bundle from node *i*, the expected recipient, for instance node *j*, utilizes ${\mathrm{AC}}_{i}$ alongside its own particular ${\mathrm{AC}}_{i}$ to decide the fitting channel and rate to be used for the information delivery.

#### 3.1.5. QoS Provisioning MAC Protocol

The QoS provisioning MAC protocol [70] was proposed by Wang et al., to overlay the Time-Division Multiple Access (TDMA) to co-exist with single-hop CRAHN. It consists of four stages of the cognition cycle, namely observe stage, plan stage, decide stage and act stage. During the observing stage, a neighbor list foundation system is acquainted with a record of the spectrum use circumstance of the PU and SU. An SU uses this list to perceive the spectrum utilization in its neighborhood.

The plan stage consist of two major functions. One is to make sure that an SU is not interfering with a licensed user, and the other one is to use the free spectrum efficiently. To achieve these functionalities a contention resolution system joined with gating, straight backoff and slow down shirking is proposed to upgrade the throughput and access deferral and decency of the protocol. To guarantee agreeable QoS on the built-up ad hoc connection, an invited reservation strategy is presented in the chosen stage; to advise users of the initial time of the Contention-Free Period (CFP) and a Contention Period (CP) in each round. The act stage includes the distributed frame synchronization mechanism for SU.

### 3.2. Time-Slotted Protocols

These MAC protocols divide time into slots, offering an inherent collision-free scheme for the control channel, as well as for the data transmission. Several of these protocols are based on Time Division Multiple Access (TDMA) and need network-wide synchronization. Similar to the random access protocol, time slotted MAC for CRAHN is also investigated in the literature. For example, energy efficient cognitive radio multichannel (ECR-MAC) [71] uses explicit frequency negotiation and time negotiation to achieve power savings by having an idle node go to sleep mode. The cognitive-radio-based multi-channel MAC (CRM-MAC) protocol [72] exploits the use of double transceivers to improve performance. In the following subsections, we analyze some of the time-slotted MAC protocols in more detail.

#### 3.2.1. Decentralized Predictive MAC Protocol

The decentralized Predictive MAC protocol (P-MAC) [73] is a synchronized MAC protocol for multi-channels and multi-handsets. A dedicated CCC is assumed to be available, and each CR device is furnished with dual half-duplex transceivers, called the switch and the information transceivers. To determine in the whole network the spectrum opportunity, P-MAC executes distributed sensing. Time synchronization is employed to delay or to stop the auxiliary transmission and decides the system-wide spectral occurrences. P-MAC utilizes the Timer Synchronization Function (TSF), similar to the IEEE 802.11 MAC protocol, for harmonization. P-MAC uses an innovative algorithm for channel selection, which depends on historical prediction model (HPM). In P-MAC, the identifying data are shared by the nodes and conserve the information of the state of the channels in the entire system for sensing and to keep the rights of the PUs.

**Figure 5:**
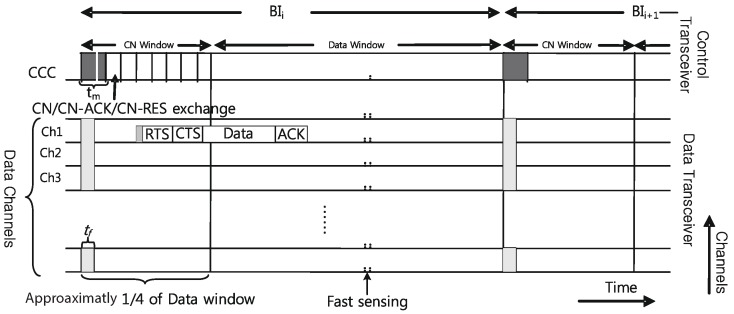
P-MAC time-channel domain structure. RES, REServation.

Similar to IEEE 802.22, P-MAC combines two sensing techniques: quick sensing, which takes little time and has low exactness, and fine sensing, which enhances precision, diminishes the rate of false caution and expands the QoS. The data transceiver is additionally in charge of fine detecting and quick detecting. The data transceiver stays in the snooze state at points where there is no information to send and receive. To conserve more energy, the transceiver stays active for 60% of the time and goes to sleep mode for the remaining 40%. Figure 5 shows the P-MAC time-channel area structure.

#### 3.2.2. Channel-Aggregation Diversity-Based MAC Protocol

Channel-Aggregation Diversity-based MAC protocol (CAD-MAC) [74] is centered on a novel differing qualities innovation called Channel-Aggregation Diversity (CAD). CAD permits the auxiliary node to use various channels in parallel for data transmission that are not being in use by PUs. It can productively distribute the upper-limited power resource for the chosen channels in view of the channel qualities through joint power-channel allocation and information radio. CAD-MAC offers two mutual power-channel provision schemes; one is the ideal allocation strategy through dynamic programming to maximize the transmission rate, and the other is a corresponding allocation strategy, which uses fractional programming for optimizing energy efficiency. In view of the joint power channel assignment approach, the node can use the channel to transmit many information packets in one transmission round.

**Figure 6:**
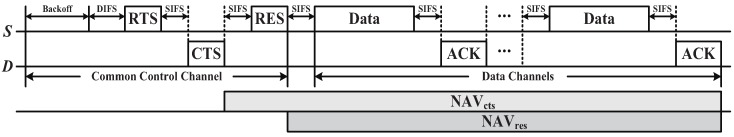
CAD-MAC transmission process.

CAD-MAC utilizes the surely understood ON/OFF model to portray PUs’ channel-utilization patterns, and all information channels are grouped into synchronized channel slots. CAD-MAC assumes all of the subsequent nodes in the CRAHN are synchronized and work with the same division of time as that of PUs to productively shield PUs from being meddled by SUs. In the CAD-MAC protocol, node pairs negotiate to access the opportunistic data channels through control packets in the transmission period, which are RTS, CTS and REServation (RES), respectively. As can be seen from Figure 6, after a successful three-way handshake (RTS-CTS-RES), data channels and rates for transmission can be determined. Then, the origin-destination pair can transmit using the selected data channels.

#### 3.2.3. Concurrent Access MAC Protocol

Concurrent Access MAC (CA-MAC) [75] tries to achieve low access delay by creating several communication pairs concurrently, which are able to start transmission simultaneously. Each node has two radio front-ends for control signals and data transmission. Therefore, control overhearing and data transmission are concurrently possible. In CA-MAC, each channel has a unique time slot, and each channel is accessed only in a time slot associated with channels.

CA-MAC uses two different channel lists, which are sorted according to priority, one is the Sorted Channel List (SCL), and the second is the Common Channel List (CCL). SCL is an arranged list of all of the channels available to members, which is based on accessibility, while CCL is a local list of channels available between a communication pair. CA-MAC uses the SCL to choose the data channel in a way that it should be a channel that is most common to all of the members. In contrast, CA-MAC uses the least common channel from CCL for the data channel between a communication pair to lower the chance of collision. In each time slot, all of the nodes set their listening radio to the associated channel. The sender chooses the first free channel from the CCL and sends the channel reservation request. Once the request is received, during the signaling period, the receiver checks the channel. When the channel is being used, the receiver suggests the next free channel from CCL. Otherwise, it sends the acknowledgment packet to the sender. By overhearing the channel reservation between a pair, other nodes update their available channel information, so they do not attempt to use the reserved channel.

#### 3.2.4. Cluster-Based MAC Protocol

**Figure 7:**
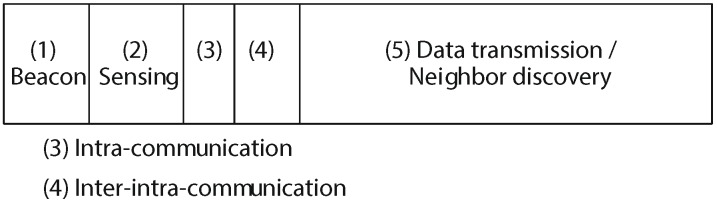
CB-MAC superframe structure.

Li et al. [76] proposed a cluster-based MAC protocol for CRAHNs, which aimed to be more robust to the PU activities to utilize the free spectrum more efficiently. Channel access time is distributed into superframes; see Figure 7. The beacon time is issued by the Cluster-Head (CH), which has time synchronization information for cluster control, cluster ID, the CH and the channel hopping sequence. Intra-cluster communication and inter-cluster communication are done through the CH.

In addition to this, a database that restores spectrum occupancy history is introduced. For each origin-destination pair, CH defines access scheduling of the channel. Nodes not participating in the communication tune to different frequencies making decisions using the experience-based channel hopping sequence to perform neighbor discovery. Using this method, the proposed cluster-based MAC protocol does not need a specific period for neighbor discovery.

The proposed MAC protocol forms a cluster based on node geographical position, available channel and experienced statistic. During the first round of neighbor discovery, information from the neighbors is collected, and a value is assigned to links to those neighbors. At the subsequent round of neighbor disclosure, nodes share their link value. Later, according to these values, nodes start forming clusters. After cluster formation, each member in the cluster is controlled by the cluster head.

#### 3.2.5. A MAC Protocol for Cognitive Radio Wireless Ad Hoc Networks

A collision-free multichannel protocol is proposed in [77] for wireless ad hoc networks. It considers QoS as a main parameter for single-hop scenarios. The resource sharing technique approves the optimized circulation of time slots between nodes. The technique is capable of improving the assignment of communication resources by considering the QoS requirements. This assignment gives more data transfer capacity and decreases delay to access resources for users with more execution necessities. The proposed protocol uses time slots and impact free, as an entrance window is given in each slot. Slots are divided into mini-slots where the nodes exploit them by using reservation with a specific end goal to attempt each one in turn to get to the accessible channel.

It is assumed that all of the nodes are synchronized, and the time is partitioned into periods comprising control time, sensing period and time slots for transmission. The control time frame is utilized to exchange notification in the network about nodes that are leaving or joining, so that every node can overhaul, as its ID needs to be. IDs determine the node access request to the network. In particular, the access window is separated into mini slots, and the highest number of mini-slots is compelled by the length of the time slot and the entrance window.

#### 3.2.6. Cognitive MAC

**Figure 8:**
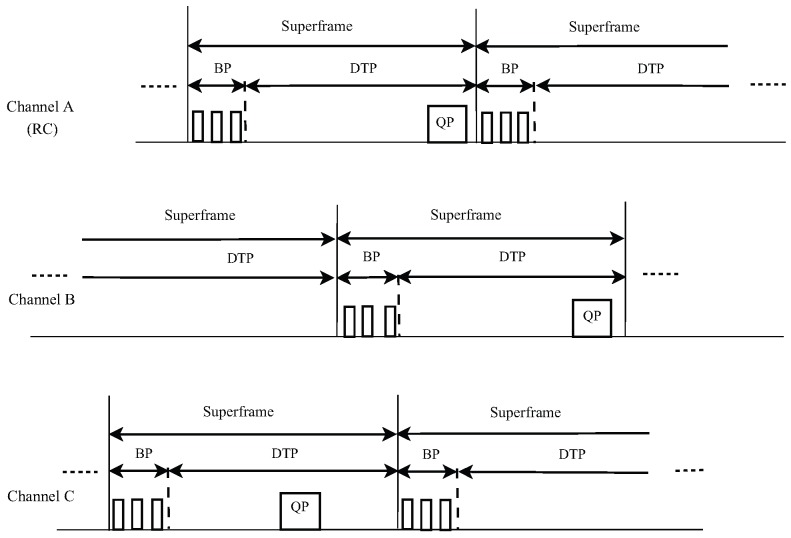
C-MAC superframe structure. RC, Rendezvous Channel; DTP, Data Transfer Period; BP, Beacon Period.

Another distributed multi-channel protocol is Cognitive MAC (C-MAC) [78], which uses multi-transceivers, synchronization and time slots. In this protocol, channels are logically organized into superframes that include a Beacon Period (BP) and a Data Transfer Period (DTP); see Figure 8. To avoid interference, each BP is additionally divided on the basis of time; thus, nodes avoid interference and exchange information by using the negotiation of channel usage.

For node coordination in different channels, among all available channel, a Rendezvous Channel (RC) is chosen dynamically and in a way that channels can be utilized for a very long time all through the network, without intrusion. The RC channel is also used for PU detection, for the interchange of the programs for the BP, as well as neighbor discovery and network-wide communication. C-MAC also uses the concept of Backup Channel (BC), i.e., if there should arise an occurrence of the presence of a PU, it instantly makes a decision of substitute spectrum bands.

The CR user starts with identifying vacant spectrum resources. In the case of hearing a beacon in any of the available bands, CR may adapt to global RC specified in the beacon and join that specific band. Moreover, the CR node informs its neighbor about other devices by re-broadcasting the beacon packet. Before using any band, and for resynchronization, the CR user needs to tune to RC periodically and transmit its beacon information and all of the changes that occurred in the spectrum. To differentiate PUs from the CR user in each spectrum, C-MAC employs non-overlapping Quiet Periods (QP). In addition, C-MAC uses node traffic reservation information by collecting the load measurements from the analysis of beacons to balance the load in the network.

### 3.3. Hybrid Protocols

Hybrid protocols are partially synchronized and partially random. In he general approach, control signaling follows over the corresponding time slots where the data communication scheme might be based on random channel access. In another approach, we might have a superframe with predefined durations that is shared with every user in the network, while the channel access may be completely random in each control or data duration.

#### 3.3.1. MAC Protocol for Spectrum-Agile Wireless Networks

The MAC protocol for spectrum-agile wireless networks (OS-MAC) [79] is a cluster–based MAC protocol for CRAHN. In OS-MAC, the author assumed the presence of a CCC where inter-channel control traffic, as well as the clustering operation take place. On the other hand, intra-cluster communication is accomplished on the Data Channel (DC) nominated by the cluster. CR users with solitary half-duplex transceivers need to switch the use of the data frequency and the CCC. To deal with the access in both the authorized and unlicensed spectrum, CR users self-organize into clusters such that every one of the individuals from the same cluster needs to communicate with each other. The cluster leader, called the delegate, is the only active node in the cluster at any given time. The other nodes keep their radio tuned to the CCC. For accessing the spectrum band, cluster members use IEEE 802.11 Distributed Coordination Function (DCF) access mode.

The OS-MAC uses periods in time that are called Opportunistic Spectrum Periods (OSPs), which are further divided into the delegate phase, select phase and update phase. Each delegate acquires information about DCs traffic load in the update phase and transfers it at the beginning of the next OSP. Based on this information of traffic, each cluster selects a DC in the select phase. The primary node that could effectively convey a message becomes the cluster delegate, which takes place in the delegate phase. This active cluster delegate also supervises the CCC for staying alert about the spectral information of the environment. In the update phase, it sends information about traffic on its cluster to other delegates, while nodes inside its cluster keep on accessing the DC. By exchanging this information with the neighboring cluster, the delegate may change the spectrum used in the cluster.

#### 3.3.2. Partially-Observable Markov Decision Process-Based MAC

The work in [80] proposes a protocol based on Partially-Observable Markov Decision Processes (POMDP). The protocol is partly a slotted single-radio MAC protocol. It divides time into slots where the CR user concludes the sharing of information about both the data and sensing. In each slot, data transmission is performed by using CSMA/CA with RTS-CTS.

In this protocol, the partial Markov decision process is used since the network state cannot be completely seen because of sensing errors. To maximize the throughput, based on the past accumulated history of the spectrum band, the best set for long-term use is chosen for data transmission and a set for sensing. Upon successful data transmission, the decision of the channel is compensated.

The authors in [80] assumed the transmitter and receiver switch their spectrum in a synchronized manner. Thus, it does not require additional CCC for this purpose. The protocol has a main drawback by assuming that the transmissions of PUs do not change over long periods of time. Moreover, since the protocol relies on the learning about the channel selection, in the initial stage, it shows a low performance, which can later be improved by time.

#### 3.3.3. Synchronized MAC

A multi-transceiver protocol is Synchronized MAC (SYN-MAC), [81], and it works for multi-hop CRN. SYNC-MAC operation does not require the existence of CCC. Meanwhile, a dedicated radio is assigned for control signal interchange and one radio for information transmission. Time is partitioned into N slots and every slot allocated to a different data channel. One slot is used particularly for control signal exchange, while for the data channel, a given node pair should assign a suitable data channel among the existing ones.

During the network initialization state, the CR users tune their committed control radios to the channel indicated by the protocol, and nodes that desire to start an information exchange broadcast a beacon in all available channels. Neighboring nodes with interest respond with their available channel list. The transmitting node waits for the time slot corresponding to the channel that has been chosen. The given node pair then begins a settlement procedure like the IEEE 802.11 DCF [82]. Nodes that are not included in transmission process listen to channel to get flagging data or detect any possible PU activity and avoid the hidden terminal problem.

Though SYN-MAC is not using dedicated CCC and it avoids the hidden terminal problem, it fails to utilize the available channel efficiently. Moreover, since the arrival of a PU is notified to neighbors in a specific time slot, the chance of interference between PU and CR users is high.

#### 3.3.4. Opportunistic MAC Protocol

**Figure 9:**
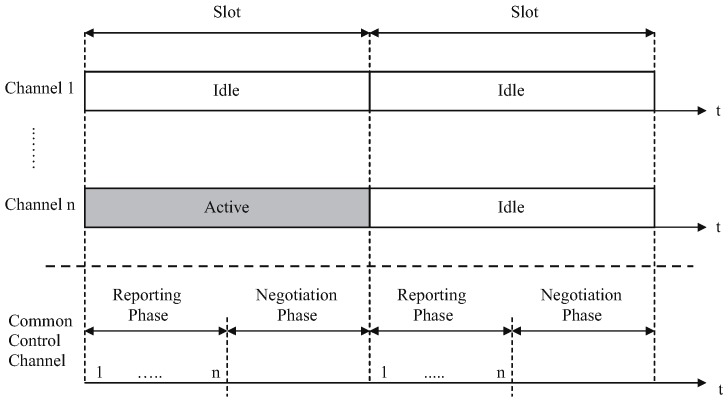
Opportunistic MAC principle.

A protocol that integrates spectrum sensing and packet scheduling is that of Su et al., in [83]. It is a protocol with multi-transceiver opportunistic cognitive MAC, and the sensing is done at the PHY layer and the scheduling at the MAC layer. One transceiver is dedicated to CCC and one to the data transmission. For the data transfer over the licensed channels, opportunistic MAC divides time into different slots. CCC is divided into two different parts. The first part is the reporting phase, and it is partly slotted. It assigns a mini-slot to each channel. Toward the start of every time slot, the CR operator detects one of the channels. In the event that any channel is observed to be unmoving, the CR user sends a reference point over the CCC during that mini-slot. The second part is the negotiation phase, which is based on random access. Figure 9 illustrates the principle of opportunistic MAC. Similar to IEEE 802.11 and *p*-persistent carrier sense multiple access, CR operators exchange via contention-based algorithms. Each CR user selects randomly and independently a channel to guarantee that all of the channels are identified. Maintaining time synchronization places extra overhead on the protocol. Moreover, since the channel for sensing is arbitrarily selected, the neighboring nodes do not have the earlier information of this occasion and do not quiet their own transmissions to enhance the detecting accuracy, which is a disadvantage of this protocol.

## 4. Discussion

Table 1 shows the reviewed MAC protocols and their main features. The MAC protocol framework for CRAHN is still an open research issue. Even though the literature has several papers published in this area, several open issues have not been fully explored. We briefly discuss some of these in the following.

### 4.1. Cross-Layer Design Has to Be Considered

In CRAHN, integrating the information from multiple layers in the MAC protocol can bring various benefits to the network. For example, the MAC layer can use the PHY layer information about channel sensing and interference detection to gradually build a model of the channel occupancy history. Channel sensing has been integrated with MAC in [83], but other actions such as interference detection and collaboration at the PHY layer can be useful. Although this model may vary with location and time of the day, it can help MAC protocols to update the strategy for selection of frequencies and the corresponding transmission. Assuming the channel occupancy model, the CR user can queue packets to be communicated through the off periods of duty-cycled PUs, which can help the CR user to conserve energy and also reduce the chance of interference with PUs.

### 4.2. Efficient Topology Management Is Required

In CRAHNs, different users experience a non-uniform channel availability and diverse PU movement in their regions. Thus, sensing results in different sets of available channels and the functions of topology discovery and management become difficult tasks. Apart from this, in distributed CRAHN, the CR users monitoring the earlier channel and waiting for the length expected to finish the information exchange on the possessed connection before starting their own transmission are ignorant of the spectrum change on that particular link, which leads to inefficient spectrum use. Therefore, having some sort of synchronization mechanisms among the CR users would help to have a more efficient network with better end-to-end performance. Clustering can be an option where the cluster head can coordinate different CR users in each cluster. It may also facilitate better cooperative spectrum sensing schemes.

### 4.3. Spectrum and User Mobility Have to Be Considered Jointly

Apart from PU appearance or channel quality degradation due to interference, which are the main causes of spectrum mobility, user mobility also results in spectrum mobility, as well. Therefore, in CRAHN, spectrum and user mobility have to be considered jointly. Limiting packet loss and spectrum switching delay is a challenging task. Therefore, to achieve a faster spectrum switching time and to reduce the packet loss and operational overhead among each functionality, spectrum mobility must be designed in a cross-layer approach. Moreover, it is essential to have an accurate estimation of the latency incurred in the handoff of frequencies in order to achieve reliable communication and management. Flexibility in spectrum handoff frameworks is needed to exploit different switching methodologies (proactive and reactive spectrum handoffs), which show a diverse impact on the correspondence execution. Furthermore, the network environment and the requirements of applications may help to deal with spectrum mobility in a more efficient manner. If spectrum holes are used for spectrum mobility, then an efficient system to deliver channel availability information is needed by the CR devices to effectively use them.

### 4.4. Efficient Usage of Power and Energy Resources is Important

In most of the cases, researchers focus on solving the spectrum scarcity problem without considering the energy or computational cost. MAC protocols must be designed in a very effective manner to reduce power consumption, interference and usage of the computational power of the CR user devices to prolong their lifetime. For example, since spectrum sensing is a time- and power-consuming action, CR users may only sense channels with high availability for transmission, so that the sensing overhead and power consumption are reduced.

### 4.5. PU Activity Pattern Needs to Be Considered

In order to have successful spectrum access, nodes need to exchange the sensing information; thus, control signaling between two CR nodes needs to be uninterrupted by neighboring CR nodes, as well as by PU activity. On the other hand, the PU may access and use the licensed spectrum on an occasional basis, or in some cases, PUs might have specific transmission patterns, for example a television station that broadcasts and uses dedicated spectrum for a pre-determined time and duration. Therefore, in the design of a control channel transmission, scheduling and dynamic power control need to be wisely designed to adapt to the PUs’ pattern.

## 5. Conclusions

The massive penetration of wireless devices, the vision of the Internet of Things (IoT) and the proliferation of bandwidth-intensive applications make the traditional static spectrum allocation not feasible. CR as a prominent solution for this problem is introduced. Several cognitive functionalities directly depend on the MAC protocol performance such as spectrum sharing and allocation and spectrum mobility. This paper presents a comprehensive summary of the state-of-the-art MAC protocols for CRAHN where fundamental issues and key considerations in MAC protocols have been critically discussed. Before discussing different MAC protocols, cognitive radio cycle and functionality have been presented for a better understanding of the spectrum management process. MAC protocols are classified based on their medium access scheme, and in each category, different MAC protocols are critically discussed and analyzed. A comparison table of discussed MAC protocols is provided to help the reader to see the differences of each protocol at a glance. Finally, a detailed discussion of the open research issues and future research directions is provided to help the reader to understand how the research in this field is evolving and which parts need further investigation.

## Figures and Tables

**Table 1:** CRAHN MAC protocol comparison table.

	MAC Protocol	Transceiver	CCC	Hop	Multi-Channel	Main Function
**Random Access Protocols**	**CM-MAC [66]**	Single	Global	Single	Yes	Spectrum selection based on PU and SU data traffic and mobility support
	**OCC-MAC; HCC-MAC [67]**	Dual	Dedicated	Multi	No	Interference-dependent contention resolution and fairness among SU
	**AW-MAC; WFC-MAC [68]**	Single	Dedicated	Single	Yes	Using dual-receive capability of radios and doesn’t require coordination with PUs
				Multi		
	**Dual receive radio MAC [69]**	Single	Dedicated	Multi	Yes	Using dual-receive capability of radios
	**QoS provisioning MAC [70]**	Single	Global	Single	No	Satisfying QoS for delay-sensitive traffic
**Time Slotted Protocols**	**P-MAC [73]**	Dual	Global	-	No	Channel selection algorithm based on Historical Prediction Model (HPM)
	**CAD-MAC [74]**	Single	Dedicated	-	Yes	Allowing each node to utilize multiple channels simultaneously
	**CA-MAC [75]**	Dual	Non	Single	Yes	Allowing multiple communication pairs to transmit data concurrently at the same time on different channels
	**Cluster-based MAC [76]**	Single	Non	Single	No	Forming stable clusters by considering experience database for choosing the communication channels
	**A MAC for CRAHN [77]**	Single	Dedicated	Single	Yes	Optimize the resource assignment by taking into account the QoS requests of nodes
	**C-MAC [78]**	Single	Dedicated	Multi	Yes	Using node traffic reservation information to balance the load in the network
**Hybrid Protocols**	**OS-MAC [79]**	Single	Global	Single	Yes	Balancing the traffic load over all spectrum bands
	**POMDP based MAC [80]**	Single	Non	Multi	No	Allows easy incorporation of spectrum sensing error and constraint on the probability of colliding with the primary users
	**SYNC MAC [81]**	Dual	Non	Multi	Yes	Multi-hop support without using CCC
	**Opportunistic MAC [83]**	Dual	Dedicated	-	Yes	SUs collaboratively sense and dynamically utilize the available frequency spectrum
